# Effect of Quercetin on Paraoxonase 2 Levels in RAW264.7 Macrophages and in Human Monocytes––Role of Quercetin Metabolism

**DOI:** 10.3390/ijms10094168

**Published:** 2009-09-23

**Authors:** Christine Boesch-Saadatmandi, Renata Toedter Pospissil, Anne-Christin Graeser, Raffaella Canali, Inka Boomgaarden, Frank Doering, Siegfried Wolffram, Sarah Egert, Manfred James Mueller, Gerald Rimbach

**Affiliations:** 1 Institute of Human Nutrition and Food Science, Christian Albrechts University Kiel, Olshausenstrasse 40, D-24118 Kiel, Germany; E-Mails:ch.boesch@foodsci.uni-kiel.de (C.B.S.);renata_pospi@yahoo.de (R.T.P.);graeser@foodsci.uni-kiel.de (A.C.G.);Inka.Boomgaarden@uk-sh.de (I.B.);doering@molprev.uni-kiel.de (F.D.);s-egert@nutrfoodsc.uni-kiel.de (S.E.);mmueller@nutrfoodsc.uni-kiel.de (M.J.M.); 2 National Research Institute for Food and Nutrition (INRAN), 00178 Roma, Italy; E-Mail:canali@inran.it (R.C.); 3 Institute of Animal Nutrition and Physiology, Christian Albrechts University Kiel, 24118 Kiel, Germany; E-Mail:wolffram@aninut.uni-kiel.de (S.W.)

**Keywords:** quercetin, flavonoid, paraoxonase 2, macrophage, atherosclerosis

## Abstract

There is increasing evidence that the intracellular antioxidant enzyme paraoxonase 2 (PON2) may have a protective function in the prevention of atherogenesis. An enhancement of PON2 activity by dietary factors including flavonoids is therefore of interest. In the present study we determined the effect of quercetin on paraoxonase 2 levels in cultured murine macrophages *in vitro* and in overweight subjects with a high cardiovascular risk phenotype supplemented with 150 mg quercetin/day for 42 days *in vivo*. Supplementation of murine RAW264.7 macrophages in culture with increasing concentrations of quercetin (1, 10, 20 μmol/L) resulted in a significant increase in PON2 mRNA and protein levels, as compared to untreated controls. Unlike quercetin, its glucuronidated metabolite quercetin-3-glucuronide did not affect PON2 gene expression in cultured macrophages. However the methylated quercetin derivative isorhamnetin enhanced PON2 gene expression in RAW264.7 cells to similar extent like quercetin. Although supplementing human volunteers with quercetin was accompanied by a significant increase in plasma quercetin concentration, dietary quercetin supplementation did not change PON2 mRNA levels in human monocytes *in vivo*. Current data indicate that quercetin supplementation increases PON2 levels in cultured monocytes *in vitro* but not in human volunteers *in vivo*.

## 1. Introduction

Paroxonase 2 is an antioxidant enzyme ubiquitously expressed in human tissue including endothelial cells [1] and macrophages [2,3]. Unlike PON1, which is mainly associated with HDL, PON2 is not found in the circulation and acts as an intracellular antioxidant [2,4]. PON2-deficient mice exhibit elevated tissue levels of lipid hydroperoxides and increased migration of macrophage into the artery wall, as compared to their wildtype controls [5]. Furthermore, PON2-deficient mice develop significantly more atherosclerotic lesions than wildtype mice [5]. It is suggested that PON2 protects macrophages from foam cell formation and thus contributes to the prevention of atherogenesis [5,6]. Importantly, a decreased PON2 expression has been observed in hypercholesterolemic patients [7] and during progression of atherogenesis [8].

Quercetin is one of the major flavonoids, ubiquitously distributed in (edible) plants [9,10]. Epidemiological data suggest an inverse relation between flavonoid intake and the risk for cardiovascular disease [11]. Although there is some evidence from cell culture studies that the dietary flavonoid quercetin may induce the expression of PON1 [12], systematic studies investigating the influence of a quercetin supplementation on PON2 gene expression are missing. Therefore, studies were conducted in murine macrophages in culture as well as in human volunteers which were supplemented with quercetin and changes in PON2 gene expression and protein levels were monitored. Furthermore, we compared the PON2 inducing activity of quercetin with its metabolite quercetin-3-glucuronide (Q3G).

## 2. Results and Discussion

**Figure 1.**
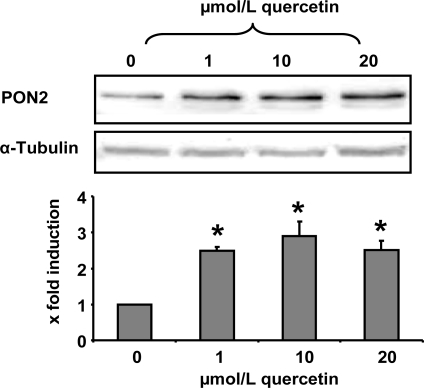
PON2 protein levels in RAW 264.7 macrophages as determined by Western blotting. Cells were treated with quercetin (0, 1, 10 and 20 μmol/L) for 24 h. The shown immunoblot is one representative out of two independent experiments. α-Tubulin was used as a loading control. * indicates significant differences (p < 0.05) in comparison to untreated control cells.

**Figure 2.**
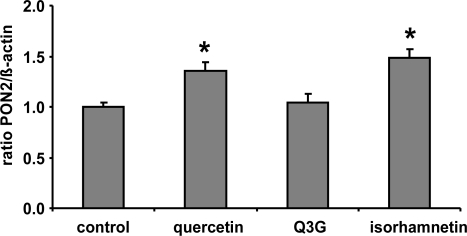
Effects of quercetin, quercetin-3-glucuronide (Q3G) and isorhamnetin on PON2 mRNA levels in RAW 264.7 macrophages. Cells were incubated 10 μmol/L of quercetin and Q3G for 6 h. Bars indicate means ± SEM of 3 experiments performed in duplicate. β-actin was used as a house-keeping gene. * indicates significant differences (p < 0.05) in comparison to untreated control cells.

In the present study we demonstrate that a quercetin supplementation up-regulates PON2 mRNA and protein levels in RAW264.7 murine macrophages in culture. As Figure 1 shows, supplementation of RAW264.7 cells with 1, 10 and 20 μmol/L quercetin resulted in a significant increase of PON2 protein levels as compared to untreated control cells. Furthermore, quercetin supplementation of RAW264.7 cells for 6 h resulted in a ~40% significant increase of PON2 mRNA levels (Figure 2).

The underlying molecular mechanisms by which flavonoids may induce PON2 gene expression in RAW264.7 cells have yet not been fully elucidated. It has been reported that flavonoids from pomegranate affect the DNA binding activity of the transcription factor AP-1 [13], which is present in the promoter region of the PON2 gene [14], thereby possibly driving PON1 gene expression. AP-1 DNA binding is partly regulated by NADPH oxidase [14]. NADPH oxidase, which produces superoxide anion free radicals, has been previously shown to be a molecular target of quercetin [15]. Furthermore, it has been recently demonstrated that the transcriptional activation of the PON2 gene is directly regulated by glucocorticoid-glucocorticoid receptor complexes, which in turn may affect the transactivation of AP-1 [16].

**Figure 3.**
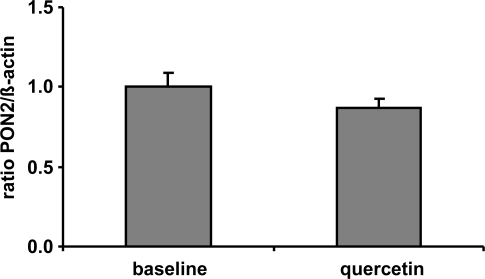
Effect of a 6-week supplementation with quercetin (150 mg/d) on PON2 mRNA levels measured in CD14-positive monocytes of human volunteers (n = 20) with a high cardiovascular risk. β-Actin was used as house-keeping gene. No significant differences between groups regarding PON2 mRNA levels were determined.

Following the assumption, that quercetin supplementation has resulted into increased PON2 mRNA and protein levels *in vitro* we supplemented human volunteers with 150 mg quercetin/day for six weeks in a placebo-controlled crossover trial (for details see [17]). In contrast to placebo, plasma quercetin concentration was significantly increased from 89.7 ± 20.7 nmol/L to 298.0 ± 30.8 nmol/L following dietary quercetin supplementation. Despite the ~3-fold increase in plasma quercetin compared to baseline quercetin levels, our data indicate that PON2 mRNA levels of human CD14 positive monocytes were not changed in response to a dietary quercetin supplementation *in vivo* (Figure 3). The plasma quercetin concentration, as measured in this human pilot study, was still lower as compared to the lowest quercetin concentration used in our cell culture experiments. The matter of fact that PON2 mRNA levels were increased in response to the quercetin supplementation in RAW264.7 cells but not in human monocytes may be related to differences in the quercetin concentrations administered *in vitro* as compared to the *in vivo* study.

Furthermore, it needs to be taken into account that following absorption in the gastrointestinal tract, quercetin is intensively metabolized leading to methylated derivatives as well as conjugates with glucuronic acid or sulphuric acid [18]. Conjugation of flavonoids including quercetin is catalysed by UDP-glucuronosyltransferases and sulfotransferases, enzymes which are present in the intestinal epithelium and in the liver [19]. The resulting compounds include quercetin monoglucuronides and monosulfates, diglucuronides and disulfates, or mixed conjugates with one site glucuronidated and one site sulphated [20,21]. The formation of conjugates converts quercetin to more water-soluble products and may affect their chemical and biological activity. Only a small portion of the free aglycone has been detected in blood, demonstrating a high rate of conjugation [22,23]. Our cell culture data suggest that quercetin but not Q3G induces PON2 (Figure 2). Glucuronidation, which masks important hydroxyl groups of the quercetin molecule, decreases its PON2 inducing activity. Thus the lack of induction of PON2 by dietary quercetin in our human study may be mainly related to the fact that most quercetin in the human circulation is present in its conjugated form. Our data for quercetin are in accordance with previous studies with genistein suggesting that sulfation of genistein, with the associated loss of hydroxyl groups, decreases its antioxidant activity and its beneficial effect on platelet aggregation, inflammation, cell adhesion and chemotaxis [24,25]. Furthermore isorhamnetin exhibited a similar PON2 inducing activity as compared to quercetin (Figure 2) indicating that methylation unlike glucuronidation of quercetin is not associated with a loss of its PON2 gene-inducing activity.

## 3. Experimental Section

### 3.1. Chemicals

Quercetin dihydrate (>98% purity) and quercetin-3-glucuronide (purity 98%) were obtained from Roth (Carl Roth GmbH, Karlsruhe, Germany). Isorhamnetin (≥95% purity) was purchased from Sigma (Deisenhofen, Germany). Cell culture medium and supplements were obtained from PAA (Coelbe, Germany). A 100 mmol/L stock was prepared with quercetin and Q3G in DMSO and stored at −20 °C until use. The primers used for the polymerase chain reaction (PCR) were obtained from MWG Biotech (Ebersberg, Germany). Western blotting reagents and materials were purchased from Bio-Rad (Bio-Rad Laboratories GmbH, Muenchen, Germany) if not otherwise stated.

### 3.2. Cell Culture and Treatments

The murine macrophage cell line RAW264.7 was purchased from DSMZ (Braunschweig, Germany). The cells were cultivated in Dulbecco’s modified Eagle medium (DMEM), high glucose, supplemented with 10% foetal bovine serum, 100 U/mL penicillin and 100 μg/mL streptomycin. Cells were grown under standard conditions in a humidified incubator at 37 °C and 5% CO_2_.

**Figure 4.**
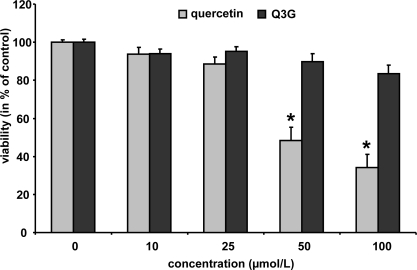
Cytotoxicity data for quercetin and quercetin-3-glucuronide. RAW264.7 cells were incubated with increasing concentrations of the test components and cytotoxicity was determined by the neutral red assay. Bars indicate means ± SEM of 3 experiments performed in triplicate. * indicates significant differences (p < 0.05) in comparison to untreated control cells.

In order to study whether the test compounds may affect cell viability, murine macrophages were incubated with increasing concentrations of quercetin and Q3G (10–100 μmol/L) for 24 h. Cytotoxicity was evaluated by neutral red assay [26]. As shown in Figure 4, quercetin did not reduce cell viability up to a concentration of 25 μmol/L; higher concentrations of 50 and 100 μmol/L reduced the cell viability to 44 and 37%, respectively. Q3G, in contrast, only slightly diminished macrophage cell viability at 50 and 100 μmol/L to 90 and 83%, respectively. Isorhamnetin did not affect cell viability at 10 and 25 μmol/L (24 h) (data not shown).

For experiments, cells were seeded at an initial density of 0.5 × 10^5^ cells per cm^2^ and incubated with quercetin in increasing concentrations (0, 1, 10 or 20 μmol/L), Q3G or isorhamnetin (10 μmol/L) for 6 h (RNA) or 24 h (Western blotting).

### 3.3. Human Study

Human monocytes originated from a subgroup (20 volunteers; 11 M, 9 F) of a recently performed quercetin-supplementation study in overweight/obese subjects (mean BMI 31.6 kg/m^2^) with high-cardiovascular disease risk phenotype at Kiel University [17]. Due to its relatively small sample size this study should be rather considered as a human pilot study. Briefly, subjects were randomised to receive 150 mg quercetin/d in a double-blinded, placebo-controlled cross-over trial with 6-week (42 days) treatment periods separated by a 5-week washout period. Participants were instructed to take a total of six capsules per day (two capsules with each principal meal). The hard gelatine capsules contained quercetin dihydrate (Voigt Global Distribution Inc. Lawrence, KS, USA), mannitol, and the flow-regulating excipient silicium dioxide. The capsules were produced at the Institute of Pharmacy, Johannes Gutenberg-University, Mainz, Germany (Dr. Peter Langguth).

Quercetin dosages were selected to represent the 15-fold the estimated daily quercetin intake in Germany of about 10 mg. Subjects were assigned to placebo or quercetin groups by blocked randomization procedure, separately for men and women. Capsules (quercetin or placebo) were handed out at days 0 and 21 of each treatment period and leftovers were collected at days 21 and 42. Compliance with treatment was 98%.

Fasting venous blood samples were taken at the first and last day of the treatment periods. Monocytes (CD14 positive) were isolated from EDTA-blood by density centrifugation with LymphoPrep™ (retailer Progen, Wieblingen, Germany) and successive positive selection with magnetic beads (Miltenyi Biotec, Bergisch Gladbach, Germany) as described previously [27]. RNA was isolated from monocytes using the Qiagen RNeasy Kit (Qiagen, Hilden, Germany).

### 3.4. RNA Isolation and Real Time PCR

Murine macrophages were lysed with TRisure reagent (Bioline, Luckenwalde, Germany) and RNA isolated according to the manufacturer’s instructions. RNA was quantified photometrically (Spectrophotometer DU800, Beckman Coulter, Krefeld, Germany). Quantitative real time PCR was performed as one step procedure using SensiMix™ One-step Kit (Quantace, Berlin, Germany) with SybrGreen detection using the Rotorgene 6000 cycler (Corbett Life Science, Sydney, Australia). Quantitation was done by use of a standard curve. Primers were designed by standard tools (Spidey, Primer3, NCBI Blast) and purchased from MWG (Ebersberg, Germany). Primer information is given below (Table 1). PON2 mRNA levels were normalized to the mRNA level of the housekeeping gene, β-actin.

### 3.5. Western Blotting

Macrophages were harvested by scraping and lysed in RIPA buffer (50 mmol/L Tris-HCl, 150 mmol/L NaCl, 0.5% deoxycholate, 0.1% sodium dodecyl sulphate (SDS), and 1% NP-40; pH 7.4 with Protease inhibitor cocktail, 1:100; Sigma, Saint Louis, USA) by incubation on ice for 30 min and subsequent centrifugation at 12,000 g (4 °C, 30 min). Protein concentration was determined in the supernantants by the BCA Assay (Pierce, Illinois, USA). 40 μg protein was separated on a 12% SDS/polyacrylamide gel and transferred onto an immunoblot polyvinylidene fluoride membrane. The membrane was blocked with 3% non-fat dried milk in Tris-buffered saline, pH 7.4, with 0.05% Tween-20 (TBS/T) for 2 h and probed with rabbit anti-PON2 antibody (1:500; abcam, Cambridge, UK) at 4 °C overnight. Then, the membranes were incubated with a goat anti-rabbit IgG secondary antibody (1:4,000) conjugated with horseradish peroxidise for 45 min. Specific bands were visualized by enhanced chemiluminescence (ECL) reagent on a ChemiDoc system and quantitated densitometrically by using the program Quantity One®. The membranes were stripped (strip buffer: 8 g glycine, 2.5 mL HCl, 1 L H_2_O) and subsequently incubated with rabbit polyclonal antibody against α-tubulin which was used as loading control (1:800, Santa Cruz Biotechnology, Heidelberg, Germany) and proceeded as described above. The predicted sizes for PON2 and α-tubulin are 40 and 55 kDa, respectively, which were checked by the use of molecular weight markers.

### 3.6. Statistical Analysis

The statistical analysis was performed with SPSS Version 15.0 (Munich, Germany). Data were tested for normal distribution (Kolmogorow-Smirnov and Shapiro-Wilk test) and analysed by t-test. In the case of non-parametric data, Mann-Whitney *U*-test was performed. Results are expressed as means with their standard errors (SEM) and significance was accepted at p < 0.05.

## 4. Conclusions

Overall, current data indicate that quercetin supplementation may increase PON2 levels in cultured murine monocytes *in vitro*, but not in monocytes of subjects exhibiting a cardiovascular risk phenotype *in vivo*. Therefore, data regarding the potential cardiovascular health benefits of flavonoids, such as quercetin, can not be directly extrapolated from an *in vitro* study, using RAW264.7 macrophages, to an *in vivo* situation, using isolated human monocytes. Furthermore our results suggest that conjugation of quercetin by glucuronic acid significantly decreases its PON2 inducing activity. Thus circulating flavonoid metabolites possess biological properties different from their nonconjugated parent compounds.

## Figures and Tables

**Table 1.** Primer sequences and conditions for real time PCR experiments.

**Gene**	**Forward Primer**	**Reverse Primer**	**Temp.**
murine β-actin	GACAGGATGCAGAAGGAGATTACT	TGATCCACATCTGCTGGAAGGT	55 °C
murine PON2	ATGGTGGCTCTGAGTTTGCT	TCCTCAGCTCCAGTTTCGAT	57 °C
human β-actin	GGATGCAGAAGGAGATCACTG	CGATCCACACGGAGTACTTG	55 °C
human PON2	TTGGACCGGCACATTTCTAT	CATGAGCCAATATGTCAGCA	55 °C
